# The Role of Anesthetic Selection in Perioperative Bleeding

**DOI:** 10.1155/2021/5510634

**Published:** 2021-05-07

**Authors:** Koichi Yuki, James A. DiNardo, Sophia Koutsogiannaki

**Affiliations:** ^1^Department of Anesthesiology, Critical Care and Pain Medicine, Cardiac Anesthesia Division, Boston Children's Hospital, USA; ^2^Department of Anaesthesia, Harvard Medical School, Boston, MA, USA; ^3^Department of Immunology, Harvard Medical School, Boston, MA, USA

## Abstract

Perioperative bleeding is one of the major comorbidities associated with surgery. While anesthesia is a critical component to perform surgery, a number of clinical studies supported the contribution of anesthetic drugs to perioperative bleeding. Here, we reviewed the literature on this topic including the underlying mechanism and discussed the future direction on coagulation research in anesthesia.

## 1. Background

Bleeding is commonly encountered during surgery. Surgical trauma directly contributes to this, and blood loss is further exacerbated by the presence of hemostatic abnormalities [1]. Hemostatic abnormalities can be congenital, acquired due to a disease process, or the result of pharmacological agent administration. The role of anesthetic agents in hemostatic changes leading to bleeding was first addressed in 1971. In a landmark paper, Ueda demonstrated that halothane attenuated platelet aggregation *in vitro* [2]. The intent of this manuscript is to review both laboratory and clinical studies investigating the effect of anesthetic agents on hemostatic function and surgical bleeding.

## 2. Clinical Studies

The majority of clinical studies investigating the effects of anesthetic agents on surgical blood loss have been prospectively randomized studies comparing volatile anesthetic agent- (isoflurane, sevoflurane, desflurane) based anesthesia to intravenous agent- (propofol) based anesthesia in patients undergoing head and neck procedures. In these studies, either surgical blood loss was directly measured or grading of surgical field visualization as a surrogate of blood loss was utilized.

Ahn et al. randomized patients undergoing endoscopic sinus surgery to either sevoflurane + remifentanil-based anesthesia (*n* = 20) or propofol + remifentanil (*n* = 20) [3]. In patients with extensive chronic sinusitis, the median blood loss of sevoflurane and propofol groups was 135 mL/hour and 19 mL/h, respectively. The surgical site visibility score was significantly worse in the sevoflurane group, indicating that propofol anesthesia offered less blood loss and better surgical field visualization. Pavlin et al. randomized patients undergoing endoscopic sinus surgery either to propofol (*n* = 30) or to isoflurane (*n* = 26) [4]. Patients in the propofol arm had better surgical field visualization by the surgical site visibility score than those in the isoflurane arm. Similarly, a number of other studies have demonstrated that propofol-based anesthesia was associated with better surgical field visualization and/or less bleeding in endoscopic sinus surgery as compared to volatile anesthetic-based anesthesia (isoflurane or sevoflurane) [5–10]. The role of anesthetics in bleeding was also examined during tonsillectomy. Okuyucu et al. randomized patients undergoing tonsillectomy to either propofol-based anesthesia (*n* = 27) or to desflurane-based anesthesia (*n* = 33) and demonstrated that propofol-based anesthesia was associated with less measured blood loss [11]. These studies indicate that an inhalation-based anesthetic technique is associated with more intraoperative bleeding than a propofol-based anesthetic technique for head and neck procedures. Law et al. randomized patients undergoing head/neck tumor resection surgery to either isoflurane-based anesthesia (*n* = 20) or propofol-based anesthesia (*n* = 18) [12]. There was no significant difference in bleeding between the groups in this study, but the median blood loss volume was higher in the isoflurane arm than in the propofol arm, consistent with other studies.

Cardiac surgery with cardiopulmonary bypass is often associated with significant perioperative bleeding [13, 14], but to date, the role of anesthetic agents in the genesis of this and other more complex surgeries has not been investigated.

## 3. Laboratory-Based Studies and Mechanistic Analysis

### 3.1. The Process of Hemostasis

Blood platelets normally flow over the vascular endothelial wall with minimal adhesion or aggregation. However, in the presence of endothelial damage, platelets are activated via a number of receptors and undergo biological changes that result in clot formation. This process can be divided into four steps: (1) adhesion of platelets to the endothelium, (2) platelet aggregation, (3) thrombin generation, and (4) contraction and clotting [15]. However, these steps do not necessarily occur sequentially and can take place simultaneously.

In damaged vessels, von Willebrand factor (vWF) and collagen are exposed within the subendothelium. Initially, platelets interact with vWF via Glycoprotein Ib (GPIb) and collagen via Glycoprotein VI (GPVI) to establish stable adhesion [16]. This leads to the activation of *α*IIb*β*3 (Glycoprotein IIb/IIIa, GPIIb/IIIa), the most abundant platelet receptor. Of note, *α*IIb*β*3 is a member of the adhesion molecule family integrins and composed of noncovalently linked *α*/*β* heterodimers. Upon activation, *α*IIb*β*3 undergoes the conformational changes that induce the ligand binding site to the high-affinity state for its ligand binding [17]. *α*IIb*β*3 in the activated conformation is able to bind to the Arg-Gly-Asp (RGD) motif-containing ligands fibrinogen and vWF (17). Once it binds to its ligands, the reorganization of actin cytoskeleton ensues (outside-in signal). Outside-in signal regulates platelet spreading over a fibrinogen surface and platelet-dependent clot retraction. Thus, the binding of fibrinogen to activated *α*IIb*β*3 is critical for clot strength. *α*IIb*β*3 is activated from intracellular signals as the result of activation of various receptors on the platelet (inside-out signal) and changes its conformation from resting conformation to activated conformation. Several soluble platelet agonists can activate *α*IIb*β*3 with varying degrees of strength. The weak agonists are epinephrine, prostaglandin E_2_ (PGE_2_), serotonin, and adenosine triphosphate (ATP). The moderate agonists are adenosine diphosphate (ADP) and thromboxane A_2_ (TXA_2_) while the strongest agonist is thrombin. Thrombin acts on platelets through protease-activated receptors (PARs) 1 and 4 [18]. Weak agonists induce minimal Ca^2+^ responses. Moderate agonists induce repetitive spiking Ca^2+^ response. Thrombin induces prolonged Ca^2+^ rise and phosphatidylserine exposure. All agonists also cause internalization of GPIb and externalization of *α*IIb*β*3 in platelets.

Thrombin formation plays a pivotal role in the development of adequate coagulation. Thrombin formation occurs in the three phases: initiation, priming, and propagation. The initiation phase begins when there is a damage to the vessel wall allowing the binding of circulating factor VIIa to tissue factor (TF) [19]. Factor X is then activated by the TF/VIIa complex leading to the formation a factor Va/Xa complex on the TF-bearing cell surface. This complex converts a small amount of prothrombin to thrombin. In addition, factor IX is activated and is an important step in the propagation phase. In the priming phase, this small amount of thrombin activates platelets at the injury site which are bound to vWF and collagen [20]. The activated platelets release and activate factor V along with activating factors XI and VIII. These three activated factors bind to the platelets. During the propagation phase, activated factor IX from the initiation phase forms a complex with activated factor VIII. This complex then activates factor X which binds to activated factor V on the platelet surface. This protected complex then provides a burst of thrombin generation. In addition to converting circulating fibrinogen to fibrin, thrombin also affects vascular permeability and serves as a chemoattractant for monocytes. Factor XIII is activated by thrombin and strengthens the clot by cross-linking fibrin molecules. It also binds to plasmin inhibitor and inhibits fibrinolysis.

### 3.2. Experimental Platform to Examine the Role of Anesthetics in Hemostasis

Activation of platelets readily leads to aggregation. Because platelet aggregation is relatively easy to quantitate in the laboratory using light transmission aggregometry (LTA), it is the most commonly used method to study the effects of anesthetic agents on platelet function [21]. More recently, platelet flow cytometry has been utilized to study platelet aggregation [22], but its use in anesthesia research is limited to surface expression analysis. Primary aggregation occurs following binding of a platelet agonist. The binding of an agonist will activate phospholipase A_2_ and release arachidonic acid. This is then converted by cyclooxygenase- (COX-) 1 to prostaglandin G_2_ (PGG_2_) and then to TXA_2_. Secondary aggregation occurs as a response to thromboxane A_2_ secreted by platelets.

Thromboelastography (TEG) is a real-time assessment of viscoelastic clot strength in whole blood [23]. This technology provides a visual assessment of clot formation and subsequent lysis under low shear conditions (0.1/sec) similar to those present in the vena cava and well below those seen in venules, large veins, and the arterial system. The relationship between the interactions of coagulation factors, inhibitors, red blood cells, platelets, and anticoagulants during clot formation and subsequent fibrinolysis is displayed. To date, the use of this technology has been limited to evaluate the effect of anesthetics on the kinetics of clot formation and dissolution.

### 3.3. The Effect of Anesthetics on Hemostasis

*(1) Halothane*. Similar to Ueda's study, Dalsgaard-Nielsen et al. demonstrated that halothane (0.49-1.25 mM) attenuated platelet secondary aggregation induced by ADP and epinephrine as well as platelet aggregation induced by TXA_2_ [24]. Patients receiving halothane-based general anesthesia also demonstrated increased bleeding time [24].

#### 3.3.1. Sevoflurane

Horn et al. examined the effect of sevoflurane in whole blood *in vitro* [25]. Whole blood exposed to 1% sevoflurane demonstrated a significantly decreased *R* time and a significantly decreased maximum amplitude (MA) or overall clot strength on TEG. Recognizing that the binding of vWF to platelet via the GPIb receptor is an initial step of hemostasis, Horn et al. also examined the effect of anesthetics on GPIb expression. They found that 1% sevoflurane exposure attenuated both ADP and thrombin mimetic TRAP-6- (PAR1 agonist peptide) induced internalization of GPIb [25]. GPIb internalization occurs along with the externalization of *α*IIb*β*3 pool as well as its activation. This result suggests that sevoflurane attenuated the activation of platelets. Similarly, in the study by Hirakata et al., sevoflurane (0.13-0.91 mM) inhibited platelet aggregation induced by ADP, epinephrine, and arachidonic acid [26]. While halothane diminished the binding of TXA_2_ to its receptor [26, 27], sevoflurane did not inhibit the binding of TXA_2_ to its receptor [26]. Hirakata et al. proposed that sevoflurane inhibited thromboxane A2 formation. However, Honemann et al. did not find that sevoflurane (0-1.3 mM) affected TXA_2_ signaling [28]. Dogan et al. also examined platelet aggregation using blood from patients receiving sevoflurane anesthesia. There was a significant reduction in platelet aggregation induced by ADP, epinephrine, collagen, and ristocetin [29].

The effect of sevoflurane on *α*IIb*β*3 expression and its activation was also examined by Horn et al. [25]. The authors found that ADP and TRAP-6 stimulation increased the surface expression of *α*IIb*β*3 in platelets by twofold and the number of activated *α*IIb*β*3 by 10-fold [25]. However, 1% sevoflurane significantly attenuated the upregulation of its expression as well as its activation. In support of this finding, Yuki et al. showed that 2% sevoflurane directly bound to *α*IIb*β*3 and attenuated its activation in the protein-based assay system [30]. The effect of sevoflurane on *α*IIb*β*3 expression was also examined *ex vivo* in patients undergoing volatile anesthetics. Liang et al. examined the upregulation of *α*IIb*β*3 expression in patients undergoing lung cancer surgery either under isoflurane or under sevoflurane anesthesia [31]. They found that the upregulation of *α*IIb*β*3 expression was less under sevoflurane than under isoflurane. They also found that sevoflurane attenuated ADP-induced platelet aggregation more than isoflurane. Other studies have demonstrated that sevoflurane attenuated platelet aggregation induced by collagen/epinephrine and collagen/ADP in surgical patients [32]. Overall, these results are consistent with the results of clinical studies supporting the association between sevoflurane-based anesthesia and increased surgical bleeding.

#### 3.3.2. Isoflurane

Hirakata et al. demonstrated that in platelet-rich plasma, isoflurane (0.28-0.84 mM) did not significantly affect platelet aggregation induced by ADP and epinephrine [26]. TXA_2_-induced platelet aggregation in platelet-rich plasma was attenuated by isoflurane only at very high concentrations (IC_50_ 15.7 mM) [27]. In contrast to this finding, Honemann et al. using oocytes transiently expressing TXA_2_ receptor demonstrated that isoflurane (IC_50_ 0.69 mM) attenuated TXA_2_ signaling [28]. In Honemann et al.'s study, rat-originated TXA_2_ receptor was transfected. Further study is needed to determine if there is a species difference in the interaction between TXA_2_ receptor and isoflurane. Dogan et al. examined platelet aggregation using blood from patients receiving isoflurane anesthesia. They found that there was no attenuation of platelet aggregation induced by ADP, epinephrine, collagen, and ristocetin [29]. *α*IIb*β*3 plays a major role in platelet aggregation. Agonist stimulation activates *α*IIb*β*3, which binds to fibrinogen in plasma that serves as a bridge between platelets. Yuki et al. examined the effect of isoflurane on *α*IIb*β*3 activation [30]. They found that 2% isoflurane attenuated the activation of *α*IIb*β*3. The photolabeling experiment showed that isoflurane is bound to the residues near the calcium-binding site that regulates the activation of *α*IIb*β*3. Based on this result, one would expect isoflurane to significantly inhibit platelet aggregation. One potential explanation is that there are receptors involved in platelet aggregation in addition to *α*IIb*β*3. However, given that *α*IIb*β*3 is the most abundant platelet receptor, other explanation should be considered. It is known that neutrophils promote thrombosis formation by shaping the rheological environment for platelet aggregation [33]. Because isoflurane has been shown to significantly attenuate neutrophil function including its binding function [34–39], it is also possible that isoflurane attenuates neutrophil-mediated thrombosis formation in surgical patients.

#### 3.3.3. Desflurane

Frohlich et al. demonstrated that desflurane (0.5 MAC) did not affect ADP-induced platelet aggregation and GPIb expression *in vitro* [40]. However, in the study by Berlet et al., 2 maximum alveolar concentrations (MAC) of desflurane attenuated ADP- and collagen-induced platelet aggregation [41]. In a study of patients undergoing a 1 MAC of desflurane-based anesthetic, Bozdogan et al. demonstrated that desflurane did not affect collagen/ADP or collagen/epinephrine-induced platelet aggregation [32].

#### 3.3.4. Xenon

While isoflurane, sevoflurane, and desflurane are halogenated ether-based volatile anesthetics, xenon is a non-ether-based volatile anesthetic. Studies in animals and humans have shown xenon to be associated with a lack of organ toxicity, cardiovascular stability, and rapid recovery characteristics [42–44]. Xenon (65%) has been shown not to affect basal or collagen/ADP or collagen/epinephrine-induced expression of GPIb [45]. Xenon also did not affect the expression of *α*IIb*β*3 in platelets stimulated by collagen/ADP or collagen/epinephrine. Based on the limited data available currently, xenon may have favorable characteristics as regards surgical bleeding. Nonetheless, to date, there are no clinical studies examining the role of xenon in surgical bleeding and xenon is not readily available for clinical use.

#### 3.3.5. Propofol

The results from *ex vivo* studies on platelet aggregation using blood from patients receiving propofol anesthesia are somewhat inconsistent. Turkan et al. examined platelet aggregation using blood from patients receiving a propofol-based anesthesia. Platelet aggregation induced by ADP, collagen, or epinephrine was not affected by propofol [46]. The estimated propofol concentration in the study was 2 *μ*g/mL (11.2 *μ*M). In contrast, Dogan et al., using a higher dose of propofol, found that there was a decrease in platelet aggregation induced by ADP, epinephrine, collagen, and ristocetin [29]. Consistent with these findings, Aoki et al. noted that propofol (13.4 *μ*g/mL = 75 *μ*M) attenuated platelet aggregation by ADP [47].

Hirakata et al. examined platelet aggregation at two propofol concentrations *in vitro* [48]. Secondary platelet aggregation induced by ADP and epinephrine was enhanced by a propofol concentration of 40 *μ*M while it was abolished by a concentration of 100 *μ*M. Primary aggregation was not affected by propofol. This suggests that propofol likely affects the formation or function of TXA_2_. Because TXA_2_-induced platelet aggregation was enhanced by 40 *μ*M and diminished by 100 *μ*M propofol, Hirakata et al. proposed that propofol attenuated cyclooxygenase activity but enhanced the interaction of the TXA_2_ receptor-mediated signal. Propofol did not affect the binding of TXA_2_ to its receptor. TXA_2_ receptor interacts with phospholipase C, which produces inositol triphosphate (IP_3_), and propofol increased IP_3_ levels. Consistent with this, De La Cruz et al. found that propofol inhibited platelet aggregation induced by ADP (IC_50_ 136 *μ*M), collagen (IC_50_ 77.8 *μ*M), or arachidonic acid (IC_50_ 71.8 *μ*M) [49]. Aoki et al. also found that propofol (5.8 *μ*g/mL = 32.5 *μ*M) inhibited platelet aggregation induced by ADP [47]. Yuki et al. demonstrated that propofol (50 *μ*M) did not affect the activation of *α*IIb*β*3 [30]. It is important to point out that in general, the clinically relevant concentration of propofol is <50 *μ*M. Finally, *in vivo* neutrophil function was not significantly affected by propofol administration [37, 38]. All these findings are consistent with the clinical observation that a propofol-based anesthesia is not associated with increased surgical bleeding as compared to an inhalation anesthetic-based anesthesia technique.

## 4. Limitation of the Current Studies and Future Direction

Overall, there is much to be done as regards the effect of anesthetic agents on hemostasis in both clinical and laboratory-based studies.

Clinical studies examining the role of anesthetics in intraoperative bleeding so far have been limited to head and neck surgeries. As reviewed above, the majority of published studies enrolled only a small number of patients in a prospectively randomized manner with the readout of either blood loss volume or surgical field visibility scored by surgeons. Despite the small patient size, the majority of studies found statistically significant difference between different anesthetic regimens; volatile anesthetic-based anesthesia was associated with worse surgical visibility score and/or blood loss compared to propofol-based anesthesia, suggesting that the choice of anesthetics could be really significant in terms of blood loss. The next step will be to examine different anesthetic regimens in other types of surgeries, particularly surgeries associated with significant blood loss such as cardiac surgery, spine surgery, and neurosurgery. In addition, the aforementioned clinical studies seldomly involved coagulation analysis. The use of various hematological assays such as TEG and platelet aggregation assay along with clinical outcome studies will be important to understand the underlying mechanism of alternation of blood loss under different anesthetics.

From the standpoint of bench research, the studies to date have focused on platelet aggregation which is only one component of the complex coagulation process, not involving the environmental tissues that platelets adhere to. The role of anesthetics in platelet-endothelium interaction should be examined in the future, which is the basis of primary hemostasis. This can be done in static assay or flow-based assay since a flow is involved in the blood vessel, thereby shear stress. Another area that is important to explore is the role of anesthetics in thrombin burst. Compared to traditional intrinsic/extrinsic coagulation cascade proposed in 1960s, a thrombin-mediated cell-based model of coagulation (initiation, propagation, and termination) described above was introduced in 2001 [50]. Whether or not different anesthetics affect the amount of thrombin generation has not been examined. Based on the study examining the effect of different anesthetics on *α*IIb*β*3 to fibrinogen binding, which can affect platelet activation, it is certainly possible that different anesthetics affect the amount of thrombin production. The role of anesthetics in fibrinolysis is also an area that has not been explored. Certainly platelets are primary cells that are involved in hemostasis. A growing literature supports that leukocytes also play a significant role in hemostasis. For example, neutrophils also bind to inflamed endothelium at the time of vascular injury. Platelet-neutrophil aggregation on the endothelium is the crucial determinant of microvascular occlusion. Adhesion molecules such as integrin *α*M*β*2 are involved in neutrophil-platelet interaction. Knowing that *α*M*β*2 can be a target of volatile anesthetic [38, 51], it is possible that anesthetics can have an impact on this aspect.

In conclusion, the current data suggest that the type of anesthetics may impact on surgical bleeding. Further clinical studies and detailed laboratory-based studies are still needed for clinical translation.

## Data Availability

All the data are available in the manuscript.

## Additional Points

*Reprint*. Please contact koichi.yuki@childrens.harvard.edu.
